# Photoacoustic gas monitoring for anesthetic gas pollution measurements and its cross-sensitivity to alcoholic disinfectants

**DOI:** 10.1186/s12871-019-0822-7

**Published:** 2019-08-09

**Authors:** Jennifer Herzog-Niescery, Thomas Steffens, Martin Bellgardt, Andreas Breuer-Kaiser, Philipp Gude, Heike Vogelsang, Thomas Peter Weber, Hans-Martin Seipp

**Affiliations:** 1 Department of Anesthesiology, Katholisches Klinikum Bochum, Ruhr-University Bochum, St. Josef Hospital, Gudrunstraße 56, 44791 Bochum, Germany; 2 Department of Life Science Engineering, University of Applied Sciences, Giessen, Germany

**Keywords:** Gas pollution, Occupational gas exposure, Photoacoustic gas monitoring, Cross-sensitivity, Isoflurane, Sevoflurane, Desflurane, Recovery time

## Abstract

**Background:**

Real-time photoacoustic gas monitoring is used for personnel exposure and environmental monitoring, but its accuracy varies when organic solvents such as alcohol contaminate measurements. This is problematic for anesthetic gas measurements in hospitals, because most disinfectants contain alcohol, which could lead to false-high gas concentrations. We investigated the cross-sensitivities of the photoacoustic gas monitor Innova 1412 (AirTech Instruments, LumaSense, Denmark) against alcohols and alcoholic disinfectants while measuring sevoflurane, desflurane and isoflurane in a laboratory and in hospital during surgery.

**Methods:**

25 mL ethyl alcohol was distributed on a hotplate. An optical filter for isoflurane was used and the gas monitor measured the ‘isoflurane’ concentration for five minutes with the measuring probe fixed 30 cm above the hotplate. Then, 5 mL isoflurane was added vaporized via an Anesthetic Conserving Device (Sedana Medical, Uppsala, Sweden). After one-hour measurement, 25 mL isopropyl alcohol, N-propanol, and two alcoholic disinfectants were subsequently added, each in combination with 5 mL isoflurane. The same experiment was in turn performed for sevoflurane and desflurane. The practical impact of the cross-sensitivity was investigated on abdominal surgeons who were exposed intraoperatively to sevoflurane. A new approach to overcome the gas monitor’s cross-sensitivity is presented.

**Results:**

Cross-sensitivity was observed for all alcohols and its strength characteristic for the tested agent. Simultaneous uses of anesthetic gases and alcohols increased the concentrations and the recovery times significantly, especially while sevoflurane was utilized. Intraoperative measurements revealed mean and maximum sevoflurane concentrations of 0.61 ± 0.26 ppm and 15.27 ± 14.62 ppm. We replaced the cross-sensitivity peaks with the 10th percentile baseline of the anesthetic gas concentration. This reduced mean and maximum concentrations significantly by 37% (*p* < 0.001) and 86% (p < 0.001), respectively.

**Conclusion:**

Photoacoustic gas monitoring is useful to detect lowest anesthetic gases concentrations, but cross-sensitivity caused one third falsely high measured mean gas concentration. One possibility to eliminate these peaks is the recovery time-based baseline approach. Caution should be taken while measuring sevoflurane, since marked cross-sensitivity peaks are to be expected.

## Background

Occupational anesthetic gas exposure is attracting growing interest, since studies have shown that low-dose exposure to volatile anesthetics (VA) increases the number of DNA strand breaks and the micronuclei frequencies in lymphocytes and epithelial cells [1, 2]. Although exposure levels of healthcare professionals have been minimized, it still is good clinical practice to reduce gas pollution ‘as low as reasonably achievable’ [3]. The Occupational Safety and Health Administration of the United States recommends the control of VA exposure by engineering controls, good work practices, use of personal protective equipment, administrative controls, and personnel exposure and environmental monitoring. The latter is especially significant, because it demonstrates the effectiveness of the gas control program and should be performed every six months [4].

Different approaches are available to evaluate the personnel’s exposure, but measurements in the individual breathing zones are commonly performed, either by time-integrated or real-time air-sampling. A dosimeter is an example for time-integrated sampling. It measures the mean pollution level, but the minimum sampling duration is > 15 min and it is not possible to get an instant feedback [4]. Hence, real-time measurements performed by gas analyzers are preferred, because they present mean and maximum concentrations continuously and enable an immediate feedback of the current VA exposure. Their low analytical detection limits allow to detect trace concentrations of 0.005 ppm. However, it is known that their accuracy may vary when they are used together with organic solvents such as alcohols [5, 6]. This is problematic for VA measurements in hospitals, because many commonly used substances contain alcohol, which may lead to a misjudgment of the actual pollution (Table 1).

**Table 1:** Commonly used disinfectants and alcoholic components which may be interfering substances while using the gas monitor. n (right column) = number of alcoholic components

Alcohol / Disinfectant	Ethyl alcohol	Isopropyl alcohol	N-propanol	Benzyl alcohol	1-tetra-decanol	Biphenyl-2-ol	Glycerol	
Hand disinfection (hygienic or preoperative)								*n*
Ethyl alcohol 80 vol.%	**X**							**1**
Isopropyl alcohol 70 vol.%		**X**						**1**
N-propanol 60 Vol.%			**X**					**1**
AHD 2000®	**X**							**1**
Aktivin® DHH		**X**						**1**
Aseptoman®		**X**						**1**
Aseptoman® viral	**X**		**X**					**2**
Aseptopur®		**X**						**1**
Descoderm®		**X**						**1**
Desderman® pure	**X**	**X**				**X**		**3**
Hospisept®	**X**		**X**					**2**
Poly-Alcohol Hands Antiseptic		**X**						**1**
Promanum pure®	**X**	**X**						**2**
Skinman® clear			**X**				**X**	**2**
Skinman® complete	**X**							**1**
Skinman® complete pure	**X**							**1**
Skinman® soft		**X**			**X**			**2**
Skinsept® F		**X**						**1**
Softa-Man®	**X**		**X**					**2**
Softa-Man® acute	**X**		**X**					**2**
Spitacid®	**X**	**X**		**X**				**3**
Sterillium®		**X**	**X**		**X**			**3**
Sterillium® classic pure			**X**		**X**			**2**
Sterillium® med	**X**		**X**		**X**			**3**
Sterillium® virugard	**X**				**X**			**2**
Skin antiseptics								**n**
Cutasept®-F, −G		**X**						**1**
Kodan® tincture forte		**X**	**X**	**X**		**X**		**4**
Octeniderm®		**X**	**X**					**2**
Poly Alcohol colourless		**X**						**1**
Surface disinfection								**n**
Amocid Lysoform®						**X**		**1**
Bacillol® AF	**X**	**X**	**X**					**3**
Mikrozid® AF liquid	**X**		**X**					**2**
Total [n]	**15**	**17**	**13**	**2**	**5**	**3**	**1**	

We investigated the cross-sensitivities of a photoacoustic gas monitor against alcohols and alcoholic disinfectants (AD) while measuring isoflurane (ISO), sevoflurane (SEVO), and desflurane (DES) in the air. Characteristic curves and an approach to deal with these interferences are presented.

We hypothesized that there is a cross-sensitivity for all three VA and that the elimination of the cross-sensitivity would reduce the average false-high VA concentration significantly by 30%. This practical impact of the cross-sensitivity was investigated on abdominal surgeons who were exposed intraoperatively to sevoflurane.

## Methods

Laboratory experiments were performed at the University of Applied Sciences, Giessen, Germany, in 2018. VA pollution measurements were conducted in a German University hospital (Katholisches Klinikum Bochum, St. Josef Hospital). The study was approved by the local ethics committee in December 2015 (No. 5184–14; Ethikkommission der Ruhr-Universität Bochum, Germany). Written informed consent was obtained from all participants included in this study. Measurements were performed according to the Declaration of Helsinki.

### Photoacoustic gas monitoring

All measurements were conducted with the photoacoustic gas monitor Innova 1412 (AirTech Instruments, LumaSense, Denmark). This analyzer identifies any gas that absorbs infrared light by using the photoacoustic infrared detection method with specific optical filters. The operating principle is as follows: A pump sucks in a sample of air into a sealed measurement cell, where pulsed infrared laser light is transferred through an optical filter. The monitored gas selectively absorbs the transmitted light, which leads to volume variations caused by increasing and decreasing temperature. This results in an acoustic signal in the measurement cell, which is proportional to the investigated gas concentration.

The gas monitor was calibrated by the manufacturer with optical filters for VA measurements (UA 0970: SEVO and DES, wavelength 8.2 μm; UA 0971: ISO, wavelength 8.5 μm) and for water vapor (SB0527, lower detection limit 50 ppm). Filters for VA measurements had a bandwidth of 6% and analytical detection limits of 0.006 ppm for SEVO, 0.005 ppm for DES and 0.005 ppm for ISO at 20 °C, 1 atm pressure and for a sample integration time of 5 s. Filters for alcohols were not used in this study to demonstrate any cross-sensitivity. A measurement interval lasted 35 s and depended on the size of the measurement cell, the length of the sampling probe, time to flush both with fresh air (30 s), and on the sample integration time (5 s). An autocalibration was performed by the gas monitor prior to each change of the VA filter. According to the manufacturer, the reproducibility of the measured anesthetic gas concentration by the photoacoustic gas monitor is ±1% of the measured value.

### Experimental setup

**Fig. 1:**
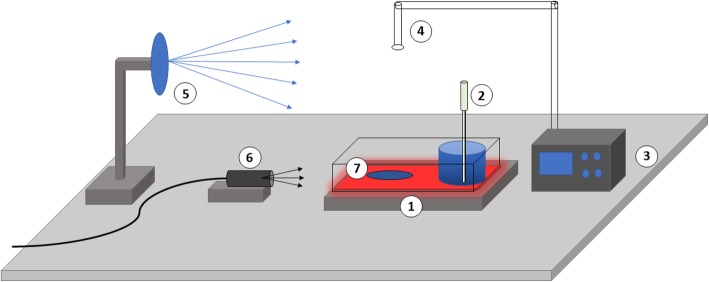
Experimental setup. A hotplate (1) was heated to 37 °C and its temperature controlled by a thermometer (2). The gas monitor’s (3) measuring probe was fixed on a stand (4). A fan was used for a uniform distribution of air (5). An Anesthetic Conserving Device was used for vaporization of the volatile anesthetic (6). The interfering agents were distributed on the hotplate (7)

The tests were conducted in a laboratory sized 36.5 m^3^. The door and the windows were kept closed. A hotplate was placed on a table and heated to 37 °C. The measuring probe of the photoacoustic gas monitor was fixed on a stand at a distance of 30 cm above the hotplate. A fan was used for a uniform distribution of air (Fig. 1).

### Baseline values

First, baseline values for ISO, SEVO and DES were determined to detect any contamination in the air. The gas monitor was calibrated to measure ISO, SEVO or DES, but neither VAs, nor interfering agents were used. A measurement interval lasted 10 min each.

### Volatile anesthetics

An Anesthetic Conserving Device (ACD; Sedana Medical, Uppsala, Sweden) was used to vaporize the VAs. Therefore, a compressed air bottle with a flow rate of 480 L/h was combined with the ACD, which was flushed with liquid VA (6 in Fig. 1). The gas monitor was equipped with the optical filter for the VA of interest. As soon as the VA concentration in the air had reached the baseline value, a bolus of 5 mL liquid VA was injected into the ACD and the gas pollution measured for one hour. Regression lines, which appear as linear curves when using a semi logarithmic diagram, were calculated after the maximum concentration was reached by the formula c = a · exp. (−b · t) with c: VA concentration [ppm]; a: factor to calculate the regression; b: gradient; t: time.

### Volatile anesthetics and interfering agents

An optical filter for ISO was used and 25 mL ethyl alcohol was distributed on the hotplate. After ten minutes of measurement, 5 ml ISO was injected into the ACD as described above, followed by a one-hour measurement. Subsequently, 25 mL isopropyl alcohol, N-propanol, AD I (isopropyl alcohol 45 vol%, N-propanol 30 vol%), and AD II (isopropyl alcohol 72 vol%) were added, each agent in combination with 5 mL ISO. Regression lines were calculated as described above.

The same tests were in turn performed with SEVO and DES.

### Approach to eliminate cross-sensitivity peaks

The approach is based on the recovery time, which is the time needed to reduce the gas concentration in the room by two log steps (corresponds to a reduction of 99%). The recovery time was calculated according to DIN EN ISO 14644 [7] and depended primarily on the type of air-conditioning, the supply air volume, the volume of the operating room, and on the position of the exhaust air slots.

To demonstrate practical significance of this approach, the gas monitor was calibrated for SEVO and the VA exposure measured during abdominal surgery in the surgeons’ individual breathing zones (25 cm around nose and mouth). The use of interfering agents was documented in time. VA concentrations and cross-sensitivity peaks were analyzed and the ‘actual’ SEVO pollution estimated.

### Statistical analysis

IBM SPSS version 20 (IBM Corp., Armonk, NY) was used for statistical analysis. After testing variables for normality using the Kolmogorov-Smirnov test and Lilliefors correction, statistical significance was determined using Students t-test and Mann–Whitney U test. Linear regression analysis was performed. Continuous variables are shown as mean (standard deviation (SD)) or median (interquartile range (IQR)). A *p*-value < 0.05 (two-sited test, error probability < 5%) was considered statistically significant.

The sample size required for measurements in hospital is based on a pilot study of 10 surgeons, who were exposed to a mean SEVO concentration of 0.58 ± 0.27 ppm including cross-sensitivity. For an α-risk of 0.05 with a power of 80% at least 16 measurements are needed to demonstrate a 30% reduction of the average SEVO concentration by elimination of the cross-sensitivity.

## Results

### Baseline values

Mean ± SD baseline values were 0.05 ± 0.01 (median: 0.05, IQR: 0.001) ppm for ISO, 0.01 ± 0.01 (median: 0.01, IQR: 0.001) ppm for SEVO, and 0.04 ± 0.01 (median: 0.05, IQR: 0.001) ppm for DES (*n* = 5 per VA).

### Volatile anesthetics

**Fig. 2:**
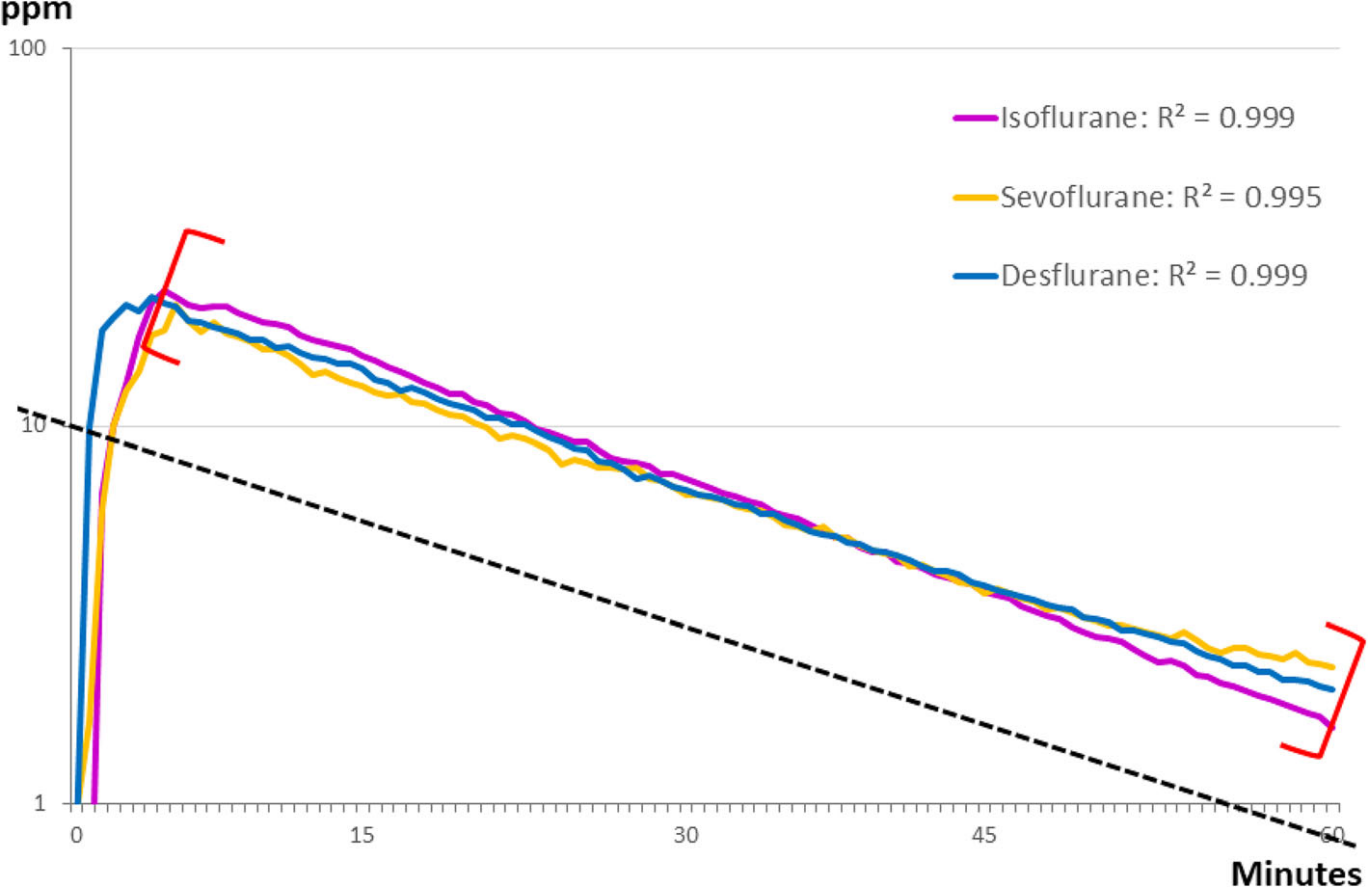
Decay curves were similar for all gases (isoflurane: y = 29.691e-0.028x; sevoflurane: y = 22.508e-0.024x; desflurane: y = 24.956e-0.025x). R^2^: exponential function with linear regression; calculations started after the maximum concentration was reached (red brackets). The recovery time was 52 min (dotted black line). *N* = 5 per anesthetic gas

The concentrations of all three VAs increased within approximately five minutes to a maximum of 21.01–23.99 ppm. The decay curves corresponded to recovery times of 52 min and exponential functions with R^2^ = 0.999 for ISO, R^2^ = 0.995 for SEVO, and R^2^ = 0.999 for DES) (n = 5 per VA) (Fig. 2).

### Volatile anesthetics and interfering agents

**Fig. 3:**
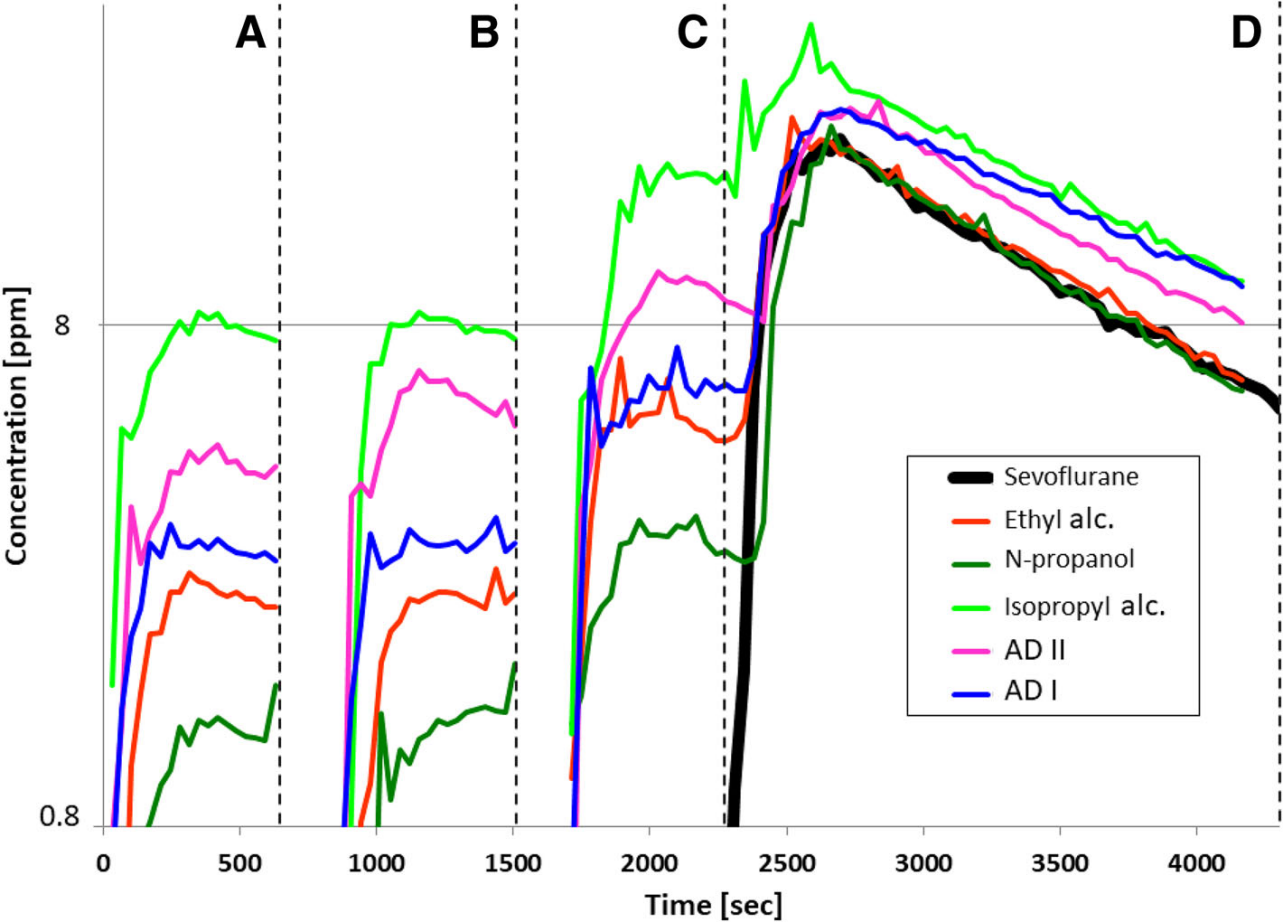
The gas monitor with filters for isoflurane (**a**), desflurane (**b**), or sevoflurane (**c**) mistakenly detects anesthetic gases, although alcohols and alcoholic disinfectants (**ad**) were used (*n* = 5 per interfering agent). The strength of the cross-sensitivity was highest for isopropyl alcohol (alc.). **d** shows the increase of the actual sevoflurane concentration (black line) by interfering agents

The gas monitor detected VA concentrations, although only alcohols and AD were used (n = 5 per interfering agent and for each VA) (Fig. 3 a-c). Consistently, highest cross-sensitivity peaks were caused by isopropyl alcohol, followed by AD, ethyl alcohol and N-propanol.

The simultaneous use of VA and interfering agent led to a false-high ‘VA concentration’ and extended the recovery time (Fig. 3d). Further, the strength of the cross-sensitivity reaction was influenced by the measured VA (SEVO > DES, ISO) (Table 2).

**Table 2:** Impact of the volatile anesthetic on the strength of the cross-sensitivity reaction for different interfering substances. Boldface entries refer to a significant value (level of significance: *p* < 0.05). * = sevoflurane caused higher false-high ‘VA’ pollution levels than desflurane or isoflurane; # = desflurane caused higher false-high ‘VA’ pollution levels than isoflurane. *N* = 5 per interfering agent and for each VA

Anesthetic gas	Desflurane					Sevoflurane				
	Ethyl alcohol	N-propanol	Isopropyl alcohol	AD I	AD II	Ethyl alcohol	N-propanol	Isopropyl alcohol	AD I	AD II
Isoflurane	**0.042#**	0.329	0.383	0.457	**< 0.001#**	< 0.001*	< 0.001*	< 0.001*	< 0.001*	< 0.001*
Sevoflurane	< 0.001*	< 0.001*	< 0.001*	< 0.001*	< 0.001*	–	–	–	–	–

### Approach to eliminate cross-sensitivity peaks

SEVO exposure in surgeon breathing zone (*n* = 20) was not distinguished between VA and interfering agents. The false-high mean and maximum SEVO concentrations were 0.61 ± 0.26 ppm and 15.27 ± 14.62 ppm, respectively.

**Fig. 4:**
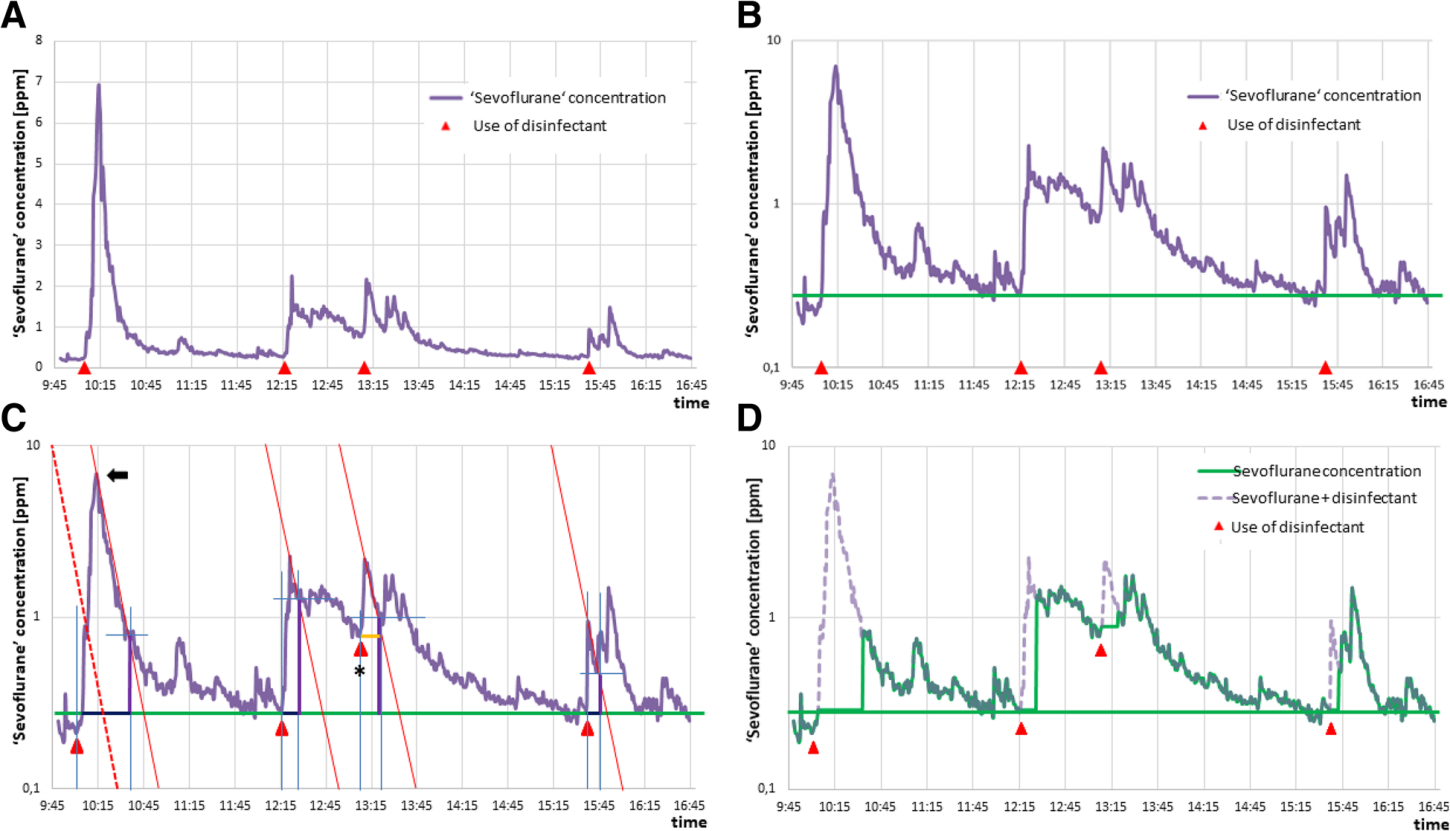
Approach to eliminate cross-sensitivity peaks. **a** shows the false-high ‘sevoflurane exposure’ of a surgeon (purple line; [ppm] mean: 0.73 ± 0.70, maximum: 6.93; red triangle: use of disinfectant). After logarithmic presentation of the data (purple line in **b**), the 10th percentile baseline is drawn (green line; here 0.29 ppm), and the recovery time is marked (red dotted line in **c**; here 39 min). This line is moved to the peak’s maximum (black arrow in **c**). The intersection with the curve marks the end of the cross-sensitivity peak (blue cross in **c**). This time interval is replaced by the baseline concentration (dark blue line in **c**). If interfering agents are used before the decay curve has reached the baseline concentration (back star in **c**) the cross-sensitivity interval should not be replaced by the 10th percentile, but by the measured concentration at the beginning of the cross-sensitivity peak (orange in **c**). In this example the ‘corrected’ mean and maximum sevoflurane concentrations were 0.53 ± 0.33 ppm and 1.76 ppm, which is a reduction by 28% (**d**)

To overcome cross-sensitivity, a 10th percentile baseline is drawn for logarithmic data with recovery time marked. A straight line is drawn from the upper left corner (0/log 10; intersection X-Y-axis) to the X-axis and moved to the peak’s maximum. The intersection with the curve marks the end of the cross-sensitivity peak. This time interval is replaced by the baseline concentration (Fig. 4).

This technique resulted in corrected mean and maximum SEVO concentrations of 0.38 ± 0.09 ppm and 0.91 ± 0.49 ppm, respectively, which is a significant reduction of 38% for mean (*p* < 0.001) and 86% for maximum (*p* < 0.001) SEVO concentrations.

## Discussion

This study investigated a photoacoustic gas monitor’s cross-sensitivities against alcohols and AD while measuring VA pollution.

In principle, photoacoustic gas monitoring is an excellent method to detect waste anesthetic gases in the air. The low detection limits allow for identification of the smallest concentrations of VAs and the immediate feedback of the VA exposure makes the gas monitor valuable in practice, because it may attract attention and help reduce the occupational gas burden. However, its cross-sensitivity to alcohols is problematic, because interfering agents are regularly used in the perioperative setting (e.g. for skin disinfection or surgical hand disinfection). This study aimed to investigate this phenomenon, since it is not considered in most clinical studies and the false-high ‘VA’ concentrations may stir unjustified fears [8–11].

The photoacoustic gas monitor’s manufacturer advises that cross-sensitivities may occur, but no information is given about the impact of the interfering agent on the measured value, the decay curve, or its significance in the clinical environment [12]. Instead, the use of additional optical filters is recommended, but this assumes that all interfering agents are known. Although it is a possible approach in an experimental setting, this is difficult in hospitals. The gas monitor can simultaneously be used with filters for the VA, water vapor, and up to four alcohols. However, this still may be insufficient as other substances interferer as well (e.g. permanent marker: ethyl alcohol and 4-chloro-3-methylphenol). Another restriction is the high costs of the optical filters. Therefore, an alternative method should be known to eliminate cross-sensitivity peaks. The presented approach can be quickly performed without technical equipment; even the recovery time can be verified in the appropriate documents for every operating room (according to ISO 14644-3 for turbulent ventilation systems) [7].

Our experiences suggest that this approach is suitable for high, as well as low cross-sensitivity peaks. Its particular value is that it considers the cross-sensitivity’s maximum, which falsely increase the mean VA concentration mostly. Each peak is replaced by the 10th percentile baseline and not just waived, which takes the actual VA concentration into account. However, it should be noted that the VA pollution during cross-sensitivity peaks is not measured but estimated. Thus, it is possible that the actual VA concentration is higher than the baseline value during a cross-sensitivity peak, which is a limitation (corrections lead to false-low VA concentrations).

In this study the elimination of the cross-sensitivity reduced the false-high mean and maximum ‘VA’ concentration by 37 and 86%, respectively. These percentages could be higher, if larger amounts of disinfectants are used [5]. This demonstrates the great impact of the cross-sensitivity on VA measurements.

Another finding was that the strength of the cross-sensitivity reaction was influenced by the measured anesthetic gas (SEVO > DES, ISO). This can be explained by the VA’s different infrared spectra, which range between 3.2 and 3.6 μm, but the absorption is highest for SEVO [12]. Consequently, it is most important to consider the gas monitor’s cross-sensitivity while measuring SEVO.

This study has a few limitations. First, we demonstrated the gas monitor’s cross-sensitivity for selected alcohols and disinfectants only, which are regularly used in hospitals. Isopropyl alcohol caused strongest peaks, however, other alcohols might show different cross-sensitivity characteristics. Further, we used 25 mL in the experimental setting only, although larger amounts are usually used in clinical routine. Much larger amounts would have increased the time to eliminate the alcohol out of the room, which correlates with increased false-high VA measurements. Nevertheless, this would not affect the approach to eliminate the cross-sensitivity peaks. Thirdly, for accuracy of the presented method, the use of every interfering agent must be documented. This might be difficult for substances, which contain ‘veiled’ interfering agents (e.g. permanent markers). Furthermore, measurements of the baseline values demonstrated that trace concentrations of interfering agents are always detectable, even in an experimental setting. Lastly, the ACD is not registered for DES, because it can boil in the agent line due to its low boiling point, which may result in an unintended emission of gas boluses [3]. However, we did not observe an uneven discharge of the anesthetic.

## Conclusions

Photoacoustic gas monitoring is an excellent method to detect trace concentrations of anesthetic gases, but clinicians are often unaware of its cross-sensitivity and overestimate VA pollution levels. This can be avoided by measurements with different optical filters or by post-hoc corrections using the described recovery time-based approach, which had reduced the false-high mean VA concentration by one third and the maximum concentration by 86% on average in this study. Caution should be taken while measuring SEVO concentrations, since marked cross-sensitivity peaks are to be expected.

## Data Availability

The datasets used and/or analyzed during the current study are available from the corresponding author on reasonable request.

## Abbreviations

ACD: Anesthetic Conserving Device
AD: Alcoholic disinfectants
DES: Desflurane
IQR: Interquartile range
ISO: Isoflurane
SD: Standard deviation
SEVO: Sevoflurane
VA: Volatile anesthetic
